# Enhanced Lightweight YOLOX for Small Object Wildfire Detection in UAV Imagery

**DOI:** 10.3390/s24092710

**Published:** 2024-04-24

**Authors:** Tian Luan, Shixiong Zhou, Guokang Zhang, Zechun Song, Jiahui Wu, Weijun Pan

**Affiliations:** College of Air Traffic Managment, Civil Aviation Flight University of China, Guanghan 618307, China; luantian@cafuc.edu.cn (T.L.); guokangzhang@cafuc.edu.cn (G.Z.); zechunsong@cafuc.edu.cn (Z.S.);

**Keywords:** wildfire detection, UAV, small-target detection, YOLOX, CSP-ML

## Abstract

Target detection technology based on unmanned aerial vehicle (UAV)-derived aerial imagery has been widely applied in the field of forest fire patrol and rescue. However, due to the specificity of UAV platforms, there are still significant issues to be resolved such as severe omission, low detection accuracy, and poor early warning effectiveness. In light of these issues, this paper proposes an improved YOLOX network for the rapid detection of forest fires in images captured by UAVs. Firstly, to enhance the network’s feature-extraction capability in complex fire environments, a multi-level-feature-extraction structure, CSP-ML, is designed to improve the algorithm’s detection accuracy for small-target fire areas. Additionally, a CBAM attention mechanism is embedded in the neck network to reduce interference caused by background noise and irrelevant information. Secondly, an adaptive-feature-extraction module is introduced in the YOLOX network’s feature fusion part to prevent the loss of important feature information during the fusion process, thus enhancing the network’s feature-learning capability. Lastly, the CIoU loss function is used to replace the original loss function, to address issues such as excessive optimization of negative samples and poor gradient-descent direction, thereby strengthening the network’s effective recognition of positive samples. Experimental results show that the improved YOLOX network has better detection performance, with mAP@50 and mAP@50_95 increasing by 6.4% and 2.17%, respectively, compared to the traditional YOLOX network. In multi-target flame and small-target flame scenarios, the improved YOLO model achieved a mAP of 96.3%, outperforming deep learning algorithms such as FasterRCNN, SSD, and YOLOv5 by 33.5%, 7.7%, and 7%, respectively. It has a lower omission rate and higher detection accuracy, and it is capable of handling small-target detection tasks in complex fire environments. This can provide support for UAV patrol and rescue applications from a high-altitude perspective.

## 1. Introduction

Forests are vital ecosystems on Earth, providing key support for biodiversity, carbon cycling, and water resources. However, forest fires, as a frequent natural disaster, not only devastate ecosystems and exacerbate climate change but also pose threats to human life and property, causing severe harm to both the ecological environment and human societal development. Protecting forests and preventing forest fires, especially by rapid and effective detection and early warning at the onset of fires, are pressing issues that need to be addressed.

The early stages of fire detection primarily relied on sensor-based methods, including smoke sensors, temperature sensors, and infrared detectors. Smoke and temperature sensors require the detection of changes in environmental smoke-particle concentration and temperature, which are limited by detection conditions and are mainly used for indoor alarms. Infrared detectors can focus on the infrared radiation of the optical unit of refraction and convert it into electrical signals, thus achieving outdoor fire alarms. For example, Le et al. [1] proposed a false-alarm-reduction system to address the problems of cumbersome traditional-manual-detection processes and high false-alarm rates. However, the above methods are affected by environmental and spatial scales, resulting in a large number of device deployments, high deployment costs, long emergency-response times, and low levels of monitoring coverage, which create certain limitations. With the development of and progress in computer vision technology, flame-detection technology has gradually evolved from sensor-based recognition to image detection and recognition, with the latter having the advantages of fast detection speed, high accuracy, and precise perception and positioning, and it has gradually become the mainstream means of fire detection. In the initial stages of image-based-mountain-fire-detection technology, machine learning algorithms were mainly used to extract and classify features such as flame color, motion, and flicker frequency and then output the flame-detection results. Ko et al. [2] proposed a fire-detection method based on visual sensors that sets the fire area by dividing the fire-moving area and its color pixels, and that combines brightness and contrast to create a brightness map, creating a time–fire model with wavelet coefficients, and that uses an SVM classifier for fire image verification, among other methods, to achieve the effective detection of continuous changes in smoke and flames. On this basis, Tom et al. [3] introduced machine learning methods using logistic regression into fire-detection work, comparing the performance of traditional fire-detection methods with machine learning-based fire-image-pixel-detection methods, providing a new direction for the development of fire-detection technology in unstructured environments. In addition, Alves et al. [4] aimed to enable early identification of mountain fires in forest environments and proposed an automated fire-detection system that uses deep convolutional neural networks (CNN) to learn fire features from 882 labeled images, achieving efficient classification of fire images with a detection accuracy of 94.1% for daytime-scene images and 94.8% for nighttime-scene target recognition, effectively reducing the false-alarm rate and missed detection rate of the model. Arul A et al. [5] proposed a machine learning-based-fire-detection system that analyzes real-time images captured by closed-circuit television (CCTV) and combines OpenCV algorithms for flame recognition, achieving early detection and warning for fires. The system integrates alarm devices and automatic-fire-extinguishing equipment, enabling rapid responses when signs of fire are detected, effectively improving the timeliness and efficiency of fire prevention and control. In recent years, with the rapid development of artificial intelligence, fire-image-detection technology based on deep learning has gradually become the mainstream, especially with the proposal and introduction of the YOLO series of models providing new ideas for mountain-fire detection. Sidhant Goyal et al. [6] fused multi-sensor monitoring signals from visible light and infrared cameras and proposed an automated early warning system based on the YOLO algorithm, using drone platforms to achieve rapid detection of forest fires, with a fire-target-detection accuracy of 90%. Li et al. [7] proposed fire-detection algorithms based on Faster-RCNN, R-FCN, SSD, and YOLOv3, respectively, with the YOLOv3-based target algorithm achieving an average precision of 83.7% and strong robustness. Wang et al. [8] proposed a lightweight forest fire-detection model based on YOLOv4, using MobileNetV3 as the backbone network, significantly reducing model parameters and improving model inference speed. Compared with the original YOLOv4, the improved model reduced the number of parameters by 62.78% and increased the inference speed by 3.04 times, providing a reference for real-time target detection of forest fires. Wu et al. [9] proposed a video fire-detection algorithm based on an improved YOLOv5 to address the limitations of traditional fire-detection methods. By introducing a dilated convolution module in the SPP module of YOLOv5, and by using GELU activation function and DIoU-NMS bounding box suppression techniques, the model significantly improves feature extraction and small-scale target detection capabilities while maintaining high detection speed. The algorithm achieves an accuracy and recall of 0.983 and 0.992, respectively, with an mAP@0.5 of 0.993 and a detection speed of 125 FPS, effectively suppressing false detections and missed detections in complex lighting environments and enhancing the robustness and reliability of the algorithm for fire detection. Zhuo et al. [10] proposed a lightweight small-target detection model, FL-YOLOv7, to address the computational capacity limitations and the balance between accuracy and computational cost of target detection models faced by unmanned aerial vehicles (UAVs) in forest fire detection. By introducing the C3GhostV2 module, SimAm attention mechanism, ASFF feature fusion module, and WIoU loss function, the algorithm improves the detection accuracy and speed of small targets such as smoke and flames while reducing model parameters and computational cost. Compared with YOLOv7, FL-YOLOv7 increases mAP50small by 2.9% and detection speed by 24.4 frames per second (FPS) while reducing the number of parameters by 27%. Talaat et al. [11] proposed an intelligent fire-detection system based on YOLOV8 for urban environment fire detection, achieving effective identification and localization of urban fires. Compared with traditional fire-detection techniques, this method can significantly improve the accuracy and speed of urban fire-target detection and significantly reduce the false-alarm rate of the algorithm, giving an accuracy and recall rate of 97.1%. In addition, YOLOX, as a lightweight model, has been widely used in the field of target monitoring since its introduction [12,13,14,15,16]. For example, Huang et al. [17] proposed a real-time forest fire-detection method called GXLD, which combines the lightweight YOLOX-L model with the dark channel-prior-defogging algorithm. By introducing GhostNet, depth-wise separable convolution, and an SE-attention mechanism, the improved algorithm can significantly reduce network parameters while improving the detection accuracy of forest fires. Experimental results show that GXLD achieves an mAP of 87.47% on the test dataset and an average frame rate of 26.33 FPS with an input image size of 1280 × 720, demonstrating its potential for the real-time and efficient detection of forest fires in complex environments. The above detection algorithms have shown significant effectiveness in fire-detection tasks and can provide effective support for fire early warning, but there is still room for improvement, mainly in the following aspects. Firstly, urban fire targets are relatively large in scale and have more distinct features than those in forest environments, making early warning highly feasible. In contrast, forest environments are more complex, including severe tree occlusion, making mountain-fire target recognition more challenging. This is particularly true in high-altitude mountainous areas, where frequent cloud and fog significantly increase the difficulty of detection. Traditional models such as YOLOX struggle to meet the requirements for real fire-target detection and early warning of mountain fires in these environments. Secondly, due to the different distances between the image acquisition equipment and the target, the varying fire intensities, and the different degrees of spread, the scale of the flames in the image varies greatly and the accuracy of multi-scale target detection needs to be improved. Thirdly, there are many types of edge-monitoring equipment with large performance differences, and the model has high computational requirements, which places a heavy burden on hardware support, which makes it difficult to effectively deploy on UAV equipment, resulting in difficulties in real-time monitoring by edge devices.

In view of this, this paper proposes a lightweight multi-scale-fire-small-target detection algorithm to provide technical support for detection and early warning in complex scenes such as forest fires. The specific contributions to this proposal include the following:

1. Designing a multi-level-feature-extraction structure CSP-ML to improve the detection accuracy of the algorithm for small-target-fire areas.
2. Optimizing the neck network structure by embedding the CBAM attention mechanism to reduce the interference caused by background noise and irrelevant information.
3. Optimizing the YOLOX network-feature-fusion mechanism by introducing an adaptive-feature-extraction module to avoid problems such as the loss of important feature information during the feature-fusion process and enhance the feature-learning ability of the network.
4. Adopting the CIoU loss function to replace the original loss function, improving the problems of excessive optimization of negative samples and poor gradient-descent direction in the original function, and strengthening the effective recognition of positive samples by the network.

The rest of this paper is organized as follows: Section 2 introduces the theoretical background of this detection method; Section 3 details the improvements in network and structure; Section 4 describes the organization and classification of the dataset and experimental results; and Section 5 discusses the results and provides conclusions.

## 2. The YOLOX Network Architecture

**Figure 1:**
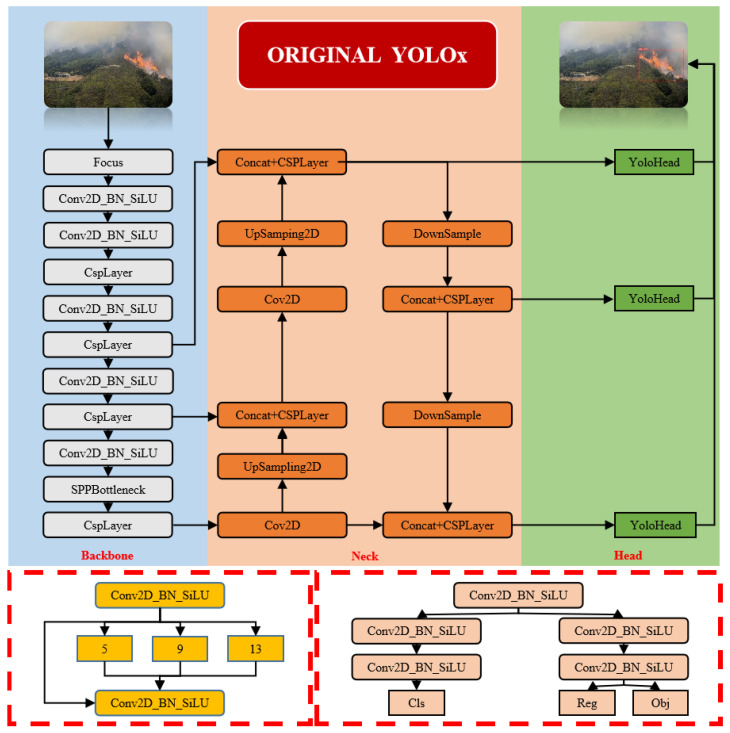
YOLOX network architecture.

Object detection is an indispensable component of computer vision, offering broad application value in practical defect identification and protective early warning tasks. YOLOX, proposed by Megvii Technology [18], is a high-performance-object-detection network designed to adapt to the demands of real-time object detection effectively, balancing detection speed and accuracy for outstanding performance in real-time applications. YOLOX inherits core concepts from the YOLO series while introducing new features and structural improvements. Compared to models such as YOLOv3, YOLOv4, and YOLOv5, YOLOX stands out in the YOLO series with its rich weight model, excellent real-time detection speed, precise detection performance, and unique decoupled-head processing approach. The YOLOX network structure is divided into four main parts: the input, the backbone for feature extraction, the neck for feature fusion, and the prediction head, as shown in Figure 1. Specifical details of this network struct are as follows:

Input: Normalizes the input images, through processes such as resizing and pixel value normalization, to prepare them for processing by the network. In some cases, it also includes data-augmentation techniques like random cropping and color adjustment to enhance the model’s generalization ability.

Backbone: Gradually compresses the image and extracts higher-level abstract features through convolutions, activation functions, and pooling layers.

Neck: Often employs structures like Feature Pyramid Networks (FPNs) or Path Aggregation Networks (PANs) to fuse feature maps of different scales. This enables the model to effectively detect targets of varying sizes, as small targets may be more easily recognized in low-level feature maps, while larger targets may be more apparent in high-level feature maps.

**Figure 2:**
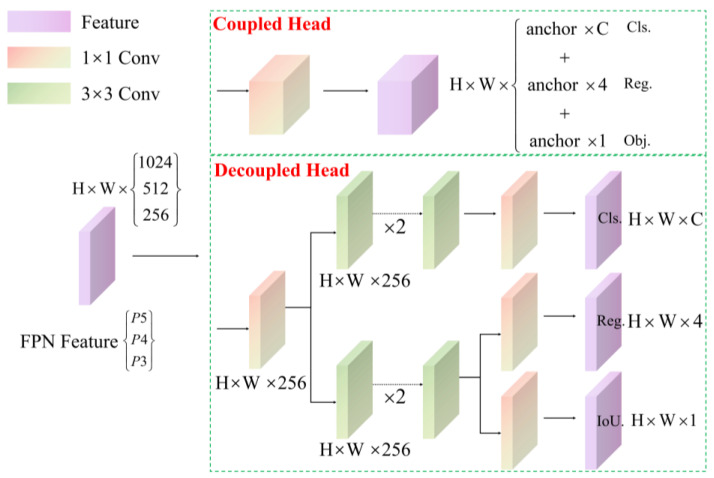
Structure of the decoupled head.

Head: Constructed with several convolutional layers, this structure includes classification and regression branches. The classification branch focuses on extracting features relevant to identifying categories through training, such as distinguishing flames and smoke from typical mountain background elements like trees and rocks, and on predicting the class of each detection box. The localization branch primarily focuses on accurately locating targets; optimizing the learning of flames and smoke in terms of size, shape, and spread to better predict their positions and extents; and retrieving the coordinates for the four points of the target boundary box. The decoupled head is shown in Figure 2. Based on the decoupling concept (separating different tasks or phases of object detection to improve model performance), this network structure design divides classification (identifying target categories) and regression (locating and sizing targets) tasks. This approach not only optimizes processing, reduces computational load, and minimizes interference between tasks, but also enhances the model’s capability in feature extraction and operational efficiency for classification. It also avoids the problems of task coupling found in traditional object-detection models where targets’ categories and positions are predicted simultaneously, potentially limiting the model’s effectiveness in complex detection scenarios. Each branch is tailored to focus on specific tasks, such as small-scale detection and high-resolution classification and localization, to enhance overall detection performance. The design of this dual-branch structure maintains the independence of tasks while implementing precise gradient adjustments and controlled backpropagation, ensuring the model’s high precision and robustness along with efficiency.

Although traditional YOLOX has demonstrated relatively superior performance in detection tasks, its application in UAV-based wildfire detection in forest and mountainous terrains faces challenges due to the complexity of fire scenes, including the following:

1. UAV aerial images cover wide areas with abundant miscellaneous information and a high proportion of small, dense targets, which complicates feature extraction such that critical fire-scene information may be overlooked by the model.
2. The background information in fire scenes is complex. In UAV images, the distribution of positive sample information, such as flames and smoke, against background elements like trees, mountains, and skies, is uneven. The original structure’s IoU loss cannot balance positive and negative samples adequately, instead over-optimizing for negative samples and neglecting positive sample recognition and severely impacting detection accuracy.

## 3. Improvements and Optimization Network

**Figure 3:**
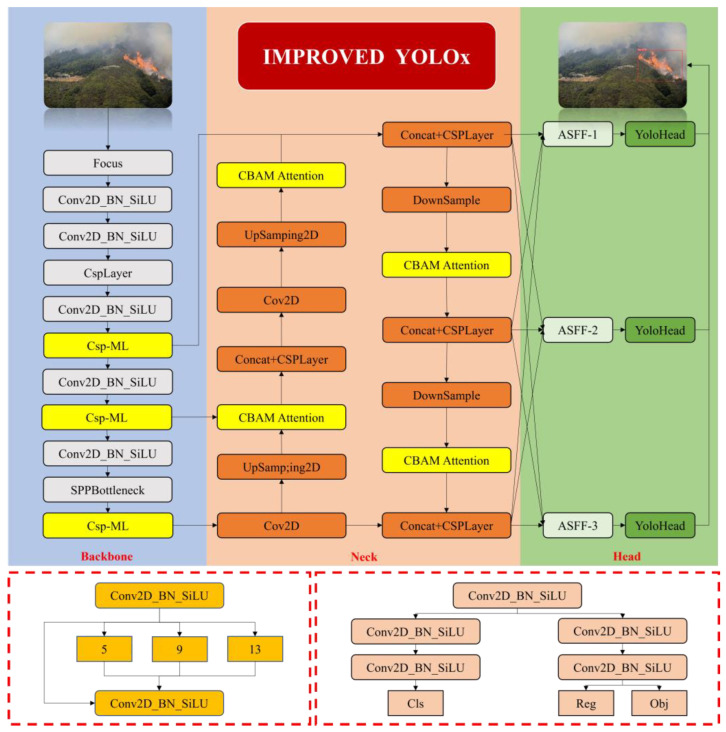
Improved YOLOX Network.

In aerial images of mountain fires captured by drones, small-sized targets such as flames and smoke are densely distributed but lack distinct features. Additionally, due to the high proportion of miscellaneous information in the environmental background, extracting effective features of critical information is challenging, resulting in low detection accuracy and a high rate of missed detections. Therefore, this paper proposes a dense small-target-wildfire-detection network based on the improved YOLOX network. The structure of this network is shown in Figure 3 and primarily consists of four parts: the input, backbone, neck, and head. To address the insufficient use of shallow-feature maps by the original network, a multi-level-feature-extraction structure, CSP-ML, is designed in the feature-extraction section to prevent the loss of semantic information contained in shallow-feature maps after multiple convolutions. Moreover, the CBAM attention mechanism is embedded in the neck of the network to precisely capture positional and channel information, which facilitates the localization of small targets. Additionally, an Adaptive Spatial Feature Fusion Module is introduced in the feature fusion section to obtain weight parameters for each feature layer, ensuring that important information predominates within feature fusion. Finally, the CIoU loss function is adopted to replace the binary IoU loss function to mitigate the impact of the numerical imbalance between target and background classes.

### 3.1. Multi-Level-Feature-Extraction Structure: CSP-ML

Multi-scale feature extraction constitutes a pivotal component within image recognition algorithms that significantly influences the algorithm’s detection accuracy and robustness. In complex wildfire scenarios, target objects frequently manifest characteristics across multiple scales and forms, which is accompanied by random occlusions. Single-scale-feature-representation methods are inadequate to fully encapsulate the intrinsic nature of the targets, resulting in decreased detection performance, missed detection, and false alarms. Therefore, the effective integration of features from multiple scales to construct scale-invariant target representations is essential for enhancing the algorithm’s capability to detect mountain fires.

The fundamental principle of multi-scale feature extraction involves extracting features characterized by complementarity and diversity from various levels and resolutions within an image, utilizing information across multiple granularities—ranging from local details to global semantics—to significantly enhance the algorithm’s adaptability to changes in target scale. With the introduction of a multi-scale analysis mechanism, image recognition algorithms are capable of capturing key features of the target across varying receptive fields. These algorithms can extract fine-grained textures, edges, and other local information while also grasping the overall structure and contextual semantics of the target, thereby facilitating precise depiction and accurate recognition of the target.

In conventional image recognition algorithms, such as Faster R-CNN, SSD, and YOLO, a multi-scale-feature-extraction strategy is extensively employed. Among these, the traditional YOLOX algorithm facilitates the cross-stage connection and integration of feature maps at differing levels through the embedding of Cross-Stage Partial (CSP) structures within various stages of the backbone network, thus enabling the capture of multi-scale fire targets.

**Figure 4:**
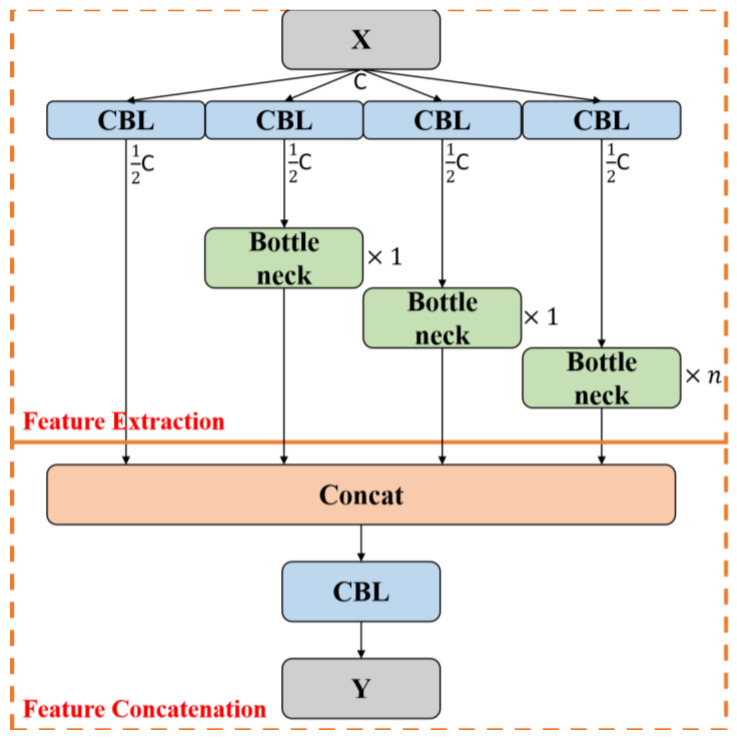
CSP-ML model architecture.

However, while the aforementioned methods facilitate the fusion of multi-scale features through cross-stage connections, their approach to fusion is relatively simplistic and overlooks the semantic interrelations between features, especially when confronted with complex mountain-fire scenarios, which potentially compromises the efficacy of feature fusion. To enhance the algorithm’s capability in recognizing and detecting multi-scale-mountain-fire regions within complex scenarios, this study introduces the concept of employing group convolution to augment feature cardinality from the ELAN model into the CSP framework, resulting in the design of a multi-level-feature-extraction mechanism, termed CSP-ML, as depicted in Figure 4.

The feature-extraction component of this structure is composed of a 1 × 1 convolution branch and three bottleneck branches. The 1 × 1 convolution branch primarily serves to diminish the number of channels in the feature map, simultaneously extracting surface location information pertaining to the fire scene such as the approximate locations of flames and smoke distribution areas. In contrast, the bottleneck branches are designed to capture deeper semantic information within the fire scene, like the extent of wildfire spread and smoke concentration, through a more profound network structure. One branch sets the number of bottleneck units to n, aiming to align the output features with the dimensions of the convolution branch outputs for straightforward subsequent concatenation, while the other two branches are equipped with only one bottleneck unit each. This asymmetrical design strategy facilitates the preservation of feature diversity and accentuates the extraction of deep-semantic information. Concatenating the feature maps from different branches along the channel dimension enables the CSP-ML structure to effectively fuse shallow-positional and deep-semantic information, thus capturing more comprehensive and accurate feature representations of complex mountain-fire scenes. The improved CSP-ML-feature-extraction process is mathematically expressed as shown in Equation (1):

(1) $$M_{b}(F)=f^{3\times 3}\left(f^{1\times 1}(F)\right)+F$$

Herein, F denotes the input feature map, and $f^{3\times 3}$ is composed of a convolution layer, a Batch Normalization (BN) layer, and an activation function. According to the formula for calculating the volume of convolutional parameters (Equation (2)), the volume of parameters in a convolutional layer is proportional to the number of input and output channels. By reducing the output channel number of the 3 × 3 convolution layer preceding the CSP structure by half and compensating for the decreased channel number with additional bottleneck branches in the CSP-ML feature-extraction structure, this approach enables effective management of the model’s parameter volume and computational cost. This balanced design ensures that, within an acceptable computational load, the model can effectively extract key features of mountain-fire scenes. The optimized CSP-ML structure, while enhancing feature-representation capabilities, also addresses the model’s need for lightness and real-time performance.

(2) $$Param_{\mathrm{conv}\;}=\left(k_{w}*k_{h}*c_{\mathrm{in}\;}\right)*c_{\mathrm{out}\;}+c_{\mathrm{out}\;}$$

In this context, $k_{w}$, $k_{h}$, $c_{in}$, and $c_{out}$ represent the width, height, number of input channels, and number of output channels of the convolution kernel, respectively. 

Outputs from the CSP-ML feature extraction process are fed into Concat, thereby facilitating multi-level feature concatenation. The optimized CSP-ML output features are detailed in Equation (3)

(3) $$F\prime =f^{3\times 3}\left(\left[M_{b}\left(f^{1\times 1}(F)\right)\right.\right.;M_{b}\left(f^{1\times 1}(F)\right);\left.\left.n*M_{b}\left(f^{1\times 1}(F)\right);f^{1\times 1}(F)\right]\right)$$

where *F* and *F*′ are the input and output feature maps, respectively.

### 3.2. Attention Mechanism: CBAM

The backbone network, as a crucial component of the YOLOx model, is primarily used to extract multi-scale feature representations from the input image and generate feature maps with different spatial resolutions (including 80 × 80, 40 × 40, and 20 × 20). These generated feature maps aggregate rich semantic information and spatial details, providing key prior knowledge for subsequent object-detection tasks. However, these feature maps also contain useless information, such as background noise. Directly concatenating and fusing them may lead to the dilution of useful information and reduce the efficiency of feature information utilization. To optimize the effect of feature fusion, researchers typically employ attention mechanisms to select and strengthen target-relevant features while suppressing background noise and irrelevant information, thereby improving the information purity of the feature maps and enhancing the model’s performance in object-detection tasks.

The more traditional attention mechanisms comprise Squeeze-and-Excitation (SE) attention, Self-Attention, Spatial Group-wise Enhance (SGE) attention, Coordination Attention (CA), ACmix attention, Spatial Attention, and Channel Attention. The Squeeze-and-Excitation (SE) attention [19] boosts the network’s capacity to discern inter-channel relations by initially compressing and then exciting the channels. This method employs global average pooling and fully connected layers to ascertain the significance of channel weights, facilitating adaptive focus on crucial features and thus enhancing the model’s performance. However, global average pooling compresses spatial details, potentially leading to a loss of essential local information. This issue might inhibit the complete capture of vital spatial data in tasks like mountain-fire detection, where backgrounds are complex and flames vary, possibly causing misses and false detections of wildfire targets. Additionally, influenced by the selection of hyperparameters, it tends to lead to overfitting and inadequate generalization capabilities of the model. Self-Attention [20], by calculating relationships among elements within the input sequence and assigning varying attention weights to each, captures long-range dependencies, making it appropriate for NLP tasks and certain imaging tasks. However, in detecting mountain fires, given the varying shapes of flames and smoke and the complex background, Self-Attention fails to adequately discern the subtle distinctions between flames and background, particularly in scenarios with small flames or dense smoke; hence, its effectiveness is limited. Spatial Group-wise Enhance (SGE) attention [21] enhances sensitivity to spatial locations by grouping feature-map channels and applying spatial attention within each group, aiding the model in better comprehending and articulating the spatial distribution among different semantic details. However, given the complex and changing environments of mountain fires, SGE’s operation with fixed group counts restricts its adaptability to various task demands, potentially increasing the computational load. Coordination Attention (CA) [22] enhances the model’s representation of local and global features through the analysis of features at various positions and their interplay. While it emphasizes the consideration of long-range dependencies across spatial and channel dimensions, its intricate coordination-relationship modeling leads to significant computational expenses and high complexity, complicating its effective deployment in edge devices for mountain-fire monitoring. The ACmix attention mechanism [23] enhances feature representation by considering both spatial and channel information, and it captures global dependencies across dimensions effectively; however, this approach is computationally complex and incurs relatively high operational costs. Spatial Attention [24] emphasizes crucial spatial areas for the current task by weighting each position differently on the feature map, yet it often overlooks the interactions and data across various channels. Channel Attention [25] concentrates on the channel dimension of the input feature maps, highlighting the channels crucial to the current task, but it may overlook the importance of spatial positions.

**Figure 5:**
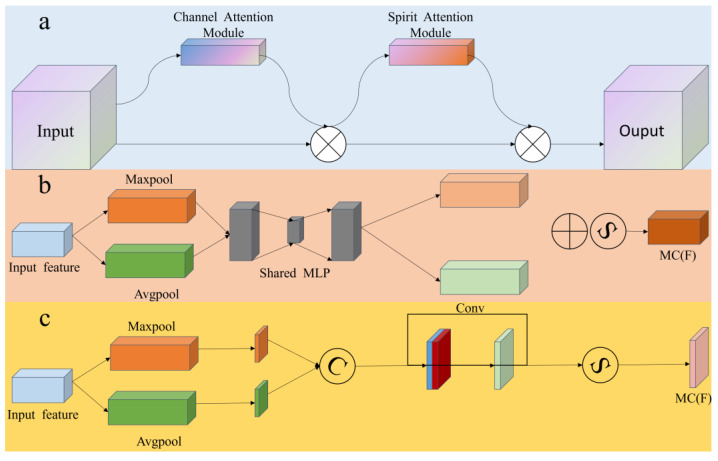
(**a**) CBAM model architecture. (**b**) Channel Attention Module (**c**) Spirit Attention Module.

The Convolutional Block Attention Module (CBAM) [26], which is extensively applied in computer vision tasks, functions by incorporating both Channel- and Spatial Attention sub-modules, adaptively modifying the significance of various channels and spatial positions within the convolutional feature map. This approach effectively diminishes disturbances from background noise and unrelated data, boosting the model’s accuracy in detecting flames and smoke, thereby enabling the network to more effectively concentrate on crucial features and their spatial details. Moreover, the implementation of CBAM incurs a minimal computational load, rendering it appropriate for use in resource-limited settings, and this provides distinct benefits for real-time or near-real-time monitoring and response to mountain fires. The CBAM attention module’s structure is depicted in Figure 5. Given the specific characteristics of mountain-fire environments and the requirements for target recognition, along with the discussed pros and cons and applicability of the attention mechanisms, the integration of the CBAM attention mechanism is considered to improve the model’s perception of mountain-fire characteristics.

The structure of the CBAM attention module is shown in Figure 4. When the feature map is input into the Channel Attention module (CAM), CAM first obtains the global information of the input feature map on each channel through global-average-pooling and global-max-pooling operations. Then, these two global-feature vectors are fed into a shared multi-layer perceptron (MLP) to generate a channel-weight vector. Finally, the weight vector is normalized to the range of {0, 1} through the Sigmoid-activation function and multiplied element-wise with the original feature map to achieve feature calibration in the channel dimension. This process can automatically identify channels that are more important and informative for the current task and assign them higher weights while suppressing redundant or irrelevant channels. Subsequently, the feature map is passed through the Spatial Attention Module (SAM), which performs average pooling and max pooling on the input feature map in the channel dimension to obtain two two-dimensional-spatial-feature maps. These two spatial-feature maps are then concatenated in the channel dimension and fed into a convolutional-layer- and Sigmoid-activation function to generate a spatial-weight map. Finally, the spatial-weight map is multiplied element-wise with the original feature map to achieve feature calibration in the spatial dimension. This process can automatically identify spatial regions that are more important and informative for the current task and assign them higher weights while suppressing the interference of background noise and irrelevant regions.

The specific working procedure is as follows:

Initially, the feature map F undergoes two parallel global pooling operations—Global Max Pooling (MaxPool) and Global Average Pooling (AvgPool)—to reduce its spatial dimensions (i.e., height and width) and obtains the global information of the input feature map on each channel. Subsequently, the output results of the pooling layer are fed into a two-layer multi-layer perceptron (MLP) to generate a channel-weight vector, which further compresses the number of channels in the feature map. Following this process, the results from the MLP are summed element-wise to obtain preliminary channel weights, which are then processed through a Sigmoid(σ) function to derive the final channel weights. Finally, the weight information is multiplied element-wise with the original feature map F to achieve feature calibration in the channel dimension, obtaining the Channel Attention-enhanced feature map $F^{\prime }$. This process can automatically identify channels that are most important and informative for the current task and assign them high weights while suppressing redundant or irrelevant channels. The process is mathematically represented as shown in Equation (4).

(4) $$F^{\prime }=F⊗\sigma (MLP(AvgPool(F)+MaxPool(F)))$$

After being enhanced by the Channel Attention mechanism, the feature map $F^{\prime }$ is further processed through global maximum pooling (MaxPool) and global average pooling (AvgPool) layers based on the channel, resulting in two channel-dimension-reduced feature maps. These compressed feature maps are then concatenated to form a combined feature map, which subsequently undergoes processing by a 7 × 7 convolution layer. Ultimately, spatial attention weights are obtained through the Sigmoid(σ) activation function. The spatial attention weight is multiplied with the Channel Attention-enhanced feature map $F^{\prime }$ to achieve feature calibration in the spatial dimension, yielding the spatial attention-enhanced feature map $F^{\prime \prime }$. This process can automatically identify spatial regions that are most important and informative for the current task and assign them high weights while suppressing the interference of background noise and irrelevant regions. The entire process is described by Equation (5) as follows:

(5) $$F^{\prime \prime }=F^{\prime }⊗\sigma \left(f^{7⊗7}\left(\left[AvgPool\left(F^{\prime }\right)\right.\right.\right.;MaxPool\left.\left.\left.\left(F^{\prime }\right)\right]\right)\right)$$

By cascading the Channel Attention module and the Spatial Attention Module, CBAM can adaptively adjust the feature map in both channel and spatial dimensions, achieving more refined and effective feature extraction. This attention mechanism has been proven to effectively enhance the representational capabilities of convolutional neural networks [27].

Considering that the background environment of mountain-fire images is intertwined with high-density flame targets, which results in complex image information, the Convolutional Block Attention Module (CBAM) is introduced after the performance of feature-layer concatenation in the neck of the YOLOx model to improve the utilization efficiency of target features and reduce the interference of irrelevant information. This deepens the network’s attention to the features of the image target region, strengthens the representation of the signature appearance features of flames and smoke in channels and spatial dimensions, and effectively suppresses the influence of background and noise factors. As a result, the improved YOLOX model can focus on the mountain-fire targets, significantly improving its detection performance and achieving precise target capture.

### 3.3. Feature Fusion: ASFF

Feature fusion is a crucial step in the object-detection process of YOLO models. The backbone network of the model extracts feature maps with different spatial resolutions and receptive fields, and these multi-scale feature maps are combined to achieve feature fusion. This process leverages the different levels of semantic information and spatial details contained in these feature maps to improve the model’s ability to detect objects of varying sizes.

Currently, the most commonly used feature-fusion modules are based on the Feature Pyramid Network (FPN) and the Path Aggregation Feature Pyramid Network (PAFPN), which enhance the model’s perception of objects at different scales through top-down and bottom-up information flow. However, these methods lack dynamic adaptation to scale variations and target size diversity in the detection task, especially in small-object detection. This static fusion strategy may lead to the loss of detailed feature information and has inherent limitations. In contrast, the Adaptive Spatial Feature Fusion (ASFF) technique addresses these issues with improvements [28]. ASFF introduces a learning-driven weight allocation mechanism that dynamically adjusts the fusion ratio of different feature maps based on the scales and complexity of the targets, prioritizing the feature information that is more beneficial to the current detection task. This fusion mechanism not only enhances the sensitivity to small objects but also provides more precise feature responses suitable for handling large-scale variations.

The working process of ASFF is as follows:

First, a convolutional layer is used to adjust the number of channels in each feature map, resizing the feature maps of different scales to a uniform scale and ensuring that they have the same number of channels. This guarantees scale invariance during feature fusion and achieves feature-map encoding. This process helps the ASFF module to better understand the contents of the feature maps and provides assistance for the subsequent weight-mapping learning.

Next, the encoded feature maps are fed into the Spatial Attention Module. The Spatial Attention Module typically consists of one or more convolutional layers, and its purpose is to learn the importance of each location in the feature map. Through convolutional operations, the Spatial Attention Module can consider both local and global information of the feature map, capture the saliency and relevance of different regions, and generate attention-weight-mapping values with the same sizes as the feature map’s α,β and γ. This weight mapping represents the importance of the corresponding location’s features. The generated attention weight mapping needs to be normalized to ensure that the weight values are within the range of 0 to 1. The normalized weight mapping represents the relative importance of each location’s features, with higher weight values indicating more important feature information at the corresponding location.

Finally, the normalized weight mapping is applied to the corresponding feature map. Through element-wise multiplication, the features are weighted and summed, which completes the adaptive fusion process. This ultimately achieves the goal of enhancing the features of important regions and suppressing the features of less important regions, resulting in the fused feature map.

**Figure 6:**
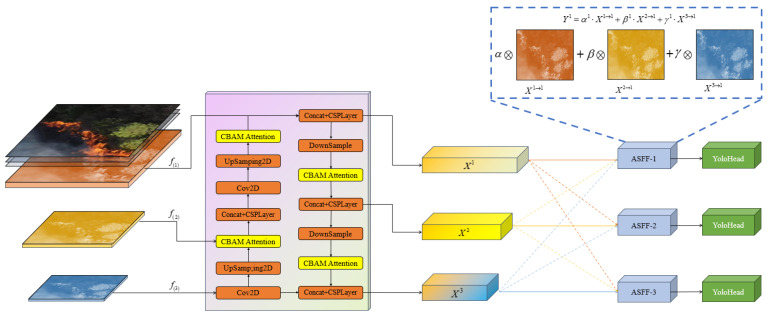
Feature fusion example utilizing ASFF-1.

In order to demonstrate the feature-fusion process more clearly, we take ASFF-1 as an example to illustrate. As shown in Figure 6, $X_{1}$, $X_{2}$, and $X_{3}$ represent feature vectors of feature maps of three scales outputted by the YOLOX path aggregation network, and we define $X^{2\to 1}$ and $X^{3\to 1}$ as feature vectors in the feature map that have been adjusted from the 2nd/3rd level to the 1st level. The feature vectors $X^{1\to 1}$, $X^{2\to 1}$, and $X^{3\to 1}$ are multiplied by their corresponding weight parameters $\alpha^{1}$, $\beta^{1}$ and $\gamma^{1}$, respectively, and then summed to output the new feature vector $Y_{1}$ that represents the feature output at the 1st level. The fusion calculation process is illustrated in Formula (6).

(6) $$Y^{1}=\alpha^{1}\cdot X^{1\to 1}+\beta^{1}\cdot X^{2\to 1}+\gamma^{1}\cdot X^{3\to 1}$$

In the equation, $\alpha^{1}+\beta^{1}+\gamma^{1}=1$, $\alpha^{1}$, $\beta^{1}$, $\gamma^{1}\in 0,1$.

Considering the advantages of ASFF in feature fusion, we replace the original PAFPN with ASFF in the YOLOx object-detection framework, optimizing the model’s feature fusion mechanism. This effectively increases the model’s utilization of small-scale object features and improves the accuracy of small-object detection. By incorporating ASFF, YOLOX is endowed with better generalization capabilities, enabling the model to adapt to more diverse object shapes and scales, making it suitable for mountain-fire-target-detection applications requiring high precision.

### 3.4. Improved Loss Function

The YOLOx loss function is typically composed of three elements: bounding box confidence loss ($L_{\mathrm{obj}}$), classification prediction loss ($L_{\mathrm{cls}}$), and bounding-box-regression loss ($L_{\mathrm{reg}}$). Specifically, components $L_{\mathrm{obj}}$ and $L_{\mathrm{cls}}$ utilize the binary cross-entropy loss function, while component $L_{\mathrm{reg}}$ employs the Intersection over Union (IoU) loss ($L_{\mathrm{IoU}}$) to gauge the accuracy of predicted box positioning. Nonetheless, if there is no intersection between the predicted and true boxes, resulting in an $L_{\mathrm{IoU}}$ value of 0, then the network may become untrainable. Additionally, a fixed $L_{\mathrm{IoU}}$ value does not guarantee a unique overlap scenario between the predicted and true boxes. Consequently, the current bounding-box-regression-loss function inadequately represents the spatial relationship and positioning accuracy between the predicted and true boxes. The calculations for IoU and IoU loss ($L_{\mathrm{IoU}}$) are defined as follows:

(7) $$R_{\mathrm{IoU}}=\frac{\left|A\cap B\right|}{\left|A\cup B\right|}$$

(8) $$L_{\mathrm{IoU}}=-\ln R_{\mathrm{IoU}}$$

To mitigate the issues impacting model robustness and enhance the localization precision of the model’s predicted bounding boxes, this study adopts CIoU loss ($L_{\mathrm{CloU}}$) as a substitute for the traditional bounding-box-regression-loss function [29]. Differing from $L_{\mathrm{IoU}}$, $L_{\mathrm{CloU}}$ evaluates not only the overlap between the predicted and actual boxes but also considers the discrepancies in the center points’ distance and aspect ratios. During training, $L_{\mathrm{CloU}}$ methodically reduces the distance between the center points of the predicted and actual boxes, thereby increasing their resemblance. This approach not only expedites network training but also refines the localization accuracy of the predicted boxes, ensuring that the model’s predicted outputs can adapt to the variable forms of targets present in fire scenarios. The CIoU calculation formula is as follows:

(9) $$L_{\mathrm{CloU}}=1-R_{\mathrm{IoU}}+\frac{d^{2}}{c^{2}}+\alpha v$$

(10) $$\alpha =\frac{v}{\left(1-R_{\mathrm{IoU}}\right)+v}$$

(11) $$v=\frac{4}{\pi^{2}}{\left(\arctan\frac{w^{\mathrm{gt}}}{h^{\mathrm{gt}}}-\arctan\frac{w}{h}\right)}^{2}$$

In these equations, d denotes the Euclidean distance between the center points of the predicted and true boxes, c refers to the diagonal length of the smallest enclosing rectangle, v quantifies the disparity in aspect ratios between the predicted and true boxes, and α serves as the weighting coefficient for v. Furthermore, w and h specify the width and height of the predicted box, respectively, while $w^{\mathrm{gt}}$ and $h^{gt}$ represent the width and height of the true box, respectively.

## 4. Experimental Results and Analysis

### 4.1. Experimental Dataset

The Southwest Forest Region, China’s second-largest natural forest area, is located in the southwestern part of the country. It primarily encompasses the areas where Sichuan, Yunnan, and Tibet intersect, including the mountainous regions of the Hengduan Mountains, the Great Bend of the Yarlung Zangbo River, and the southern foothills of the Himalayas. The area has complex terrain, high terrain, and a changeable climate. Mountain fires occur from time to time, posing a great threat to the ecological environment and the development of human society.

**Figure 7:**
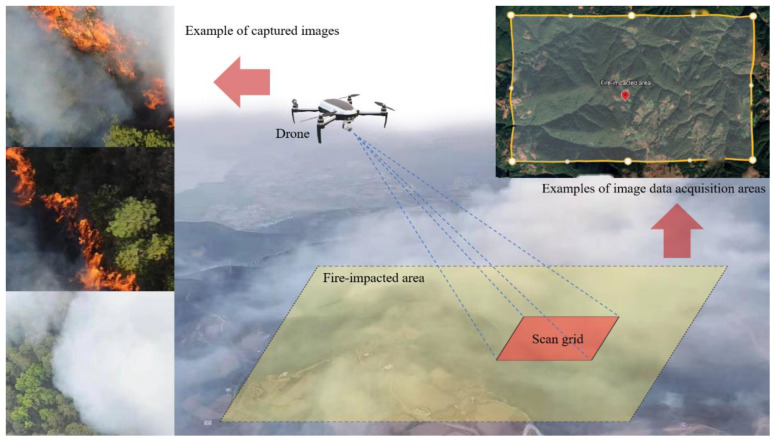
Examples of captured images.

This study targets forest mountain fire by utilizing an image dataset composed of three main parts: first, video images that were collected from on-site inspections and rescue operations during real mountain-fire incidents in parts of Sichuan, Yunnan, and other southwestern regions since 2018; second, video images from outdoor experiments simulating mountain-fire environments; and third, supplementary data from public datasets (such as FLAME and Alert Wildfire). Figure 7 presents example of images captured on-site.

Due to the randomness of mountain-fire occurrences and the variability in the environmental backgrounds of the collected images, this dataset encompasses a variety of conditions, including bright backgrounds and nighttime settings as well as scenes resembling fires and smoke. The specific distribution is as illustrated in Table 1.

**Figure 8:**
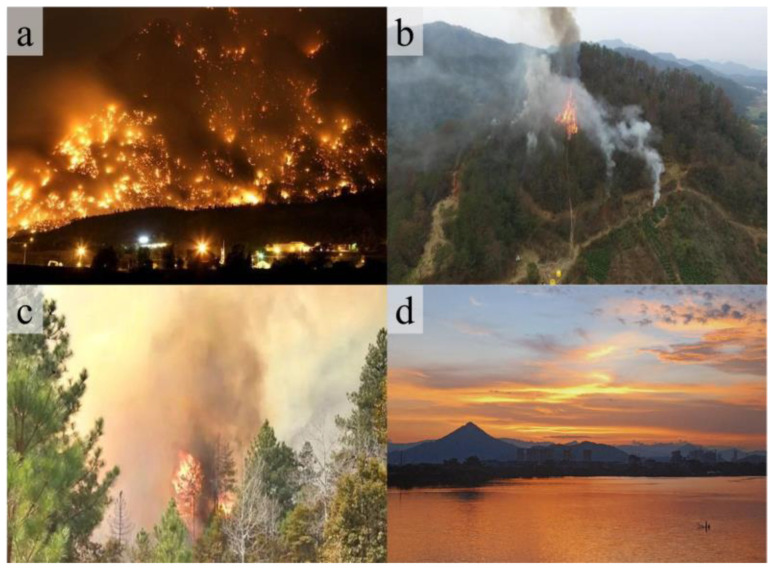
Images from the datasets used in this study (**a**–**d**).

In order to facilitate model training, all images in the data set are manually annotated with rectangular box annotations using the open source image annotation tool LABLEIMG, and corresponding JSON annotation files are generated. During the annotation process, images were categorized based on the actual conditions of fires into multiple targets, small targets, obstructed targets, and fire- or smoke-like images, with their display effects shown in Figure 8. Additionally, this experiment employs various data-augmentation techniques such as cropping, rotating, flipping, scaling, and mosaic stitching to enhance the dataset’s diversity. On this basis, a series of preprocessing steps are implemented to reduce irrelevant image information, decrease the model training’s computational requirements, and improve the trained model’s generalization performance.

The specific preprocessing steps are as follows:

1. Convert the input RGB images into single-channel grayscale images and apply Gaussian blur for filtering.
2. Randomly change the height and width of input images, allowing the dimensions of a single image to vary in multiple ratios.
3. Rotate the input images to several different angles.

Ultimately, after comparison and screening, a total of 16,817 mountain-fire-related scene images were obtained, covering 43,632 annotated points. The prepared dataset was divided into a training set (11,772 images), a validation set (3363 images), and a test set (1682 images) in a ratio of 7:2:1, which were used to train, test, and validate the effectiveness of the improved YOLOX model.

### 4.2. Experimental Environment

The model training was conducted on a Windows 10 operating system, with an NVIDIA GeForce GTX 3060 GPU (NVIDIA, Sanata Clara, CA, USA). The deep learning framework used was PyTorch 1.7.0, with Python version 3.8 and CUDA version 10.2. The experiments utilized YOLOX-s pre-trained weights, with an initial learning rate of 0.01 and a batch size of 64.

### 4.3. Evaluation Metrics

To provide a comprehensive and intuitive assessment of the improved network’s performance, this study employs metrics commonly used in object-detection tasks to evaluate the quality of model performance, such as precision, recall, F1 score, etc. The formulas and their meanings are as follows:

- *Precision*: This is a fundamental metric for measuring the performance of a model in classification tasks. It represents the ratio of correctly identified positive samples to all positive samples detected by the model. The calculation process is shown in Equation (12):

(12) $$P=\frac{TP}{TP+FP}$$

- *Recall*: *Recall* refers to the ratio of true positive samples correctly detected by the model to all positive samples in the dataset. The calculation process is illustrated as follows:

(13) $$R=\frac{TP}{TP+FN}$$

- *F1-score*: The *F1-score* is the harmonic mean of precision and recall, which serves as a comprehensive indicator of the model’s accuracy and robustness. The calculation process for the *F1-score* is as follows:

(14) $$F1-score=\frac{2\times Precision\times Recall}{Precision+Recall}$$

- *AP* (Average Precision): *AP* represents the average of precision values across all levels of recall for a specific category, reflecting the overall accuracy of the model’s detection performance on that category. The calculation process is outlined as follows:

(15) AP=∫P(R)dR

- *mAP* (mean Average Precision): *mAP* is the mean of the *AP* (Average Precision) values across all categories, and it offers a comprehensive measure of the model’s performance across all classes. If there are *N* classes, then *mAP* can be calculated as follows:

(16) $$mAP=\frac{1}{N}\sum_{i=1}^{N}AP_{i}$$

- FPS: Frame Per Second is one of the key indicators for evaluating the real-time performance of an algorithm, and it represents the number of frames an algorithm can process per second. In real-time video image processing applications, FPS directly relates to the system’s response speed and processing capability for continuous video streams or image sequences. Conventional UAV monitoring platforms or fire-monitoring towers equipped with image acquisition devices typically have a video frame rate of 30 fps. For autonomous driving or high-speed-moving-target-capture scenarios, the video fps can reach 60 fps. In engineering applications, an FPS ≥ 5 is generally sufficient to meet the recognition criteria requirements [30]. To meet the target monitoring requirements in real mountain-fire scenarios, this paper selects the higher value among the aforementioned metrics. Specifically, when the algorithm processes images with a resolution of 1200 × 800 and achieves an FPS ≥ 60, it satisfies the real-time-target-detection standard.
- Model size: Model size refers to the amount of storage space occupied by the YOLO model when stored and deployed, which is usually measured in megabytes (MB) or gigabytes (GB). It includes the model’s parameters (such as weights and biases) as well as the additional storage required for the model structure. The calculation process for model size is as follows:

(17) $$ModelSize=\sum_{i=1}^{N}\left(P_{i}\times S_{i}\right)$$

- N represents the total number of parameters in the model, $P_{i}$ represents the number of ith parameter, and $S_{i}$ represents the size of the ith parameter, usually in megabytes. For example, for a YOLO model with M convolutional layers and K fully connected layers, the number of parameters can be expressed as:
  (18) $$N=\sum_{j=1}^{M}\left(C_{in_{j}}\times C_{out_{j}}\times K_{j}\times K_{j}\right)+\sum_{k=1}^{K}\left(W_{k}\times H_{k}\right)$$

- $C_{in_{j}}$ and $C_{out_{j}}$ represent the input and output channel numbers of the jth convolutional layer, respectively. $K_{j}$ represents the convolution kernel size of the jth convolution layer, and $W_{k}$ and $H_{k}$ represent the width and height of the weight matrix of the kth fully connected layer, respectively.

Lightweight models are specifically designed and constructed as compact models that take into account model size and computational efficiency from the very beginning. These models typically have smaller numbers of parameters and simpler network structures to adapt to resource-constrained environments and real-time application requirements. In order to more precisely define the scope of lightweight models, this paper collects and analyzes the model sizes and lightweight definitions of the YOLO series models, as shown in Table 2. Through statistical analysis, it is found that most image recognition models with a model size of less than 50 MB are defined as lightweight models. Although the improved YOLOX model in this paper has a slightly larger model size and computational complexity when compared to the traditional YOLOX model that does not achieve lightweight improvement, given the early detection requirements of wildfire targets, this paper aims to maintain the lightweight-model category while improving the detection accuracy and real-time performance of the model to enable wildfire inspections using unmanned aerial vehicles (UAVs).

### 4.4. Results Analysis

#### 4.4.1. Comparison of Models’ Loss Curves

**Figure 9:**
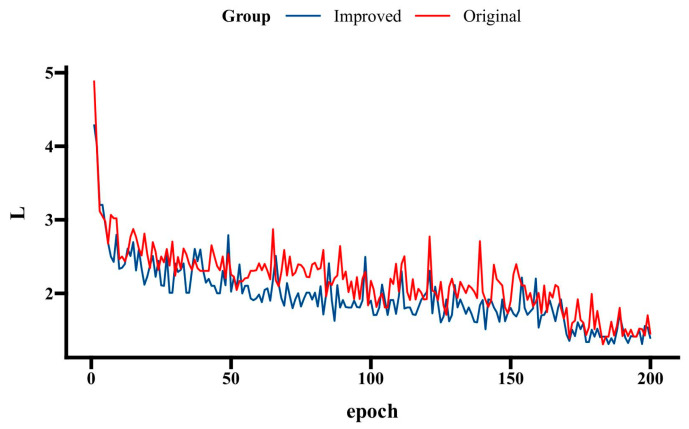
Comparison of models’ loss curves.

To verify the effectiveness of the improved YOLOX model for mountain-fire detection, an analysis and comparison of the YOLOX network’s loss curves before and after improvement were conducted, with the results depicted in Figure 9. By comparing the overall loss-curve changes, it can be observed that the improved network surpasses the original network in overall convergence speed. Particularly during the unfreezing training phase, the rate of loss reduction accelerates, tending towards a lower stable value with smaller fluctuations in the later stages. This indicates that the training of the improved YOLOX model is more effective, achieving lower error levels more quickly and with better generalization capabilities and stability. These findings validate the effectiveness of the proposed improvements.

**Figure 10:**
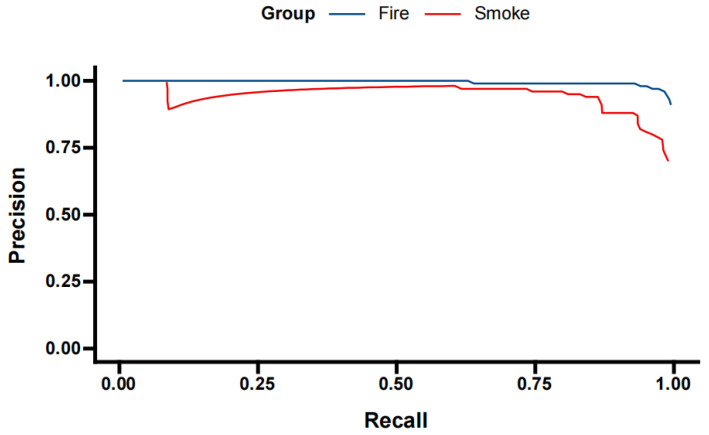
Precision–recall curve for the enhanced YOLOX model in a task detecting fire smoke.

Figure 10 illustrates the PR curve for the enhanced YOLOX model in fire-smoke-detection tasks, intuitively presenting the accuracy trends at various recall levels. For the Fire category, the model demonstrates outstanding performance throughout the entire recall spectrum. Notably, even at elevated recall levels (e.g., above 0.8), the model sustains high accuracy (approximately 0.95), indicating that it maintains effective control over false positives while detecting most real fire events, thus significantly reducing false alarms. This feature is imperative for fire early warning systems as it enables accurate detection of fire incidents promptly to secure valuable time for emergency responses. In the Smoke category, while the model’s detection efficacy marginally trails that of the Fire category, the accuracy remains approximately 0.8 at higher recall rates (e.g., above 0.7), signaling robust smoke-detection capabilities. However, with further increased recall, the Smoke category’s accuracy experiences a decline, likely due to complex environmental factors and the inherent visual diversity and ambiguity of smoke mixed with fog, which complicates detection efforts. Nonetheless, the model showcases superior performance in smoke-detection tasks, affirming the efficacy of the enhancement method.

Notably, at lower recall rates (e.g., below 0.5), the precision curves of both the Fire and Smoke categories closely align, despite minor fluctuations, and yet they remain at an exceptionally high level (nearly 1.0). This suggests that at elevated confidence thresholds the model exhibits robust discriminative capabilities for identifying fire and smoke targets, yielding highly dependable detection outcomes. However, this also suggests the potential for overlooking certain fire-smoke targets that are hard to detect. Consequently, in practical applications, detection thresholds can be dynamically adjusted based on specific requirements, striking a balance between precision and recall to optimize the model’s overall performance.

#### 4.4.2. Ablation Study

In order to more clearly analyze the impacts of each improvement module on the model’s detection performance, this paper firstly designs comparative experiments to validate the effectiveness of the CSP-ML multi-level-feature-extraction structure in enhancing the feature-extraction efforts in complex fire environments, and secondly creates four sets of ablation experiments and conducts validation experiments for 200 rounds of iterations with the same parameter settings. The results obtained are shown below:

##### Validation of the CSP-ML Multi-Level-Feature-Extraction Structure

Since the CSP-ML module focuses on extracting deep-level feature information, which hinders direct comparison, this study evaluates three distinct backbone network configurations of the YOLOX model to infer the CSP-ML module’s efficacy indirectly. Evaluated models include the original YOLOX model based on DarkNet-53, the YOLOX model utilizing ShuffleNetv2, and the YOLOX model enhanced by the CSP-ML on DarkNet-53. Comparison results, depicted in Table 3, demonstrate that the CSP-ML enhanced model outperforms the other two in terms of accuracy and recall. Notably, the CSP-ML enhanced model’s precision reaches 94.21%, surpassing the original DarkNet-53 model by 0.32 percentage points and exceeding the ShuffleNetv2 model by a substantial 6.56 percentage points. This indicates the CSP-ML-enhanced YOLOX model’s superior ability to accurately identify fire targets in images, minimizing background misclassifications. Furthermore, the model’s recall rate of 93.97%, which is 0.28% greater than that of the traditional model and 8.63 percentage points greater than that of ShuffleNetv2, underscores CSP-ML’s role in enhancing detection accuracy while maintaining comprehensive detection.

An analysis of the model’s mAP0.5 values reveals that the CSP-ML-enhanced model reached an mAP0.5 of 91.1%, marking an increase of 1.2 percentage points over the conventional model and a substantial 14.52 percentage points over the model utilizing the ShuffleNetv2 backbone network. This mAP enhancement is attributed to the multi-level structure and grouped convolutions incorporated by CSP-ML into the feature-extraction process, enabling the model to more effectively capture scale variations and detailed information of fire targets, thus achieving enhanced precision across various recall levels. This further validates the significant impact of CSP-ML in enhancing the model’s overall detection performance.

Furthermore, the F1 score—acting as the harmonic mean of precision and recall—encompasses both accuracy and comprehensiveness. The F1 score of the CSP-ML enhanced model achieved 94.51%, marking an increase of 0.54 percentage points over DarkNet-53 and a significant 15.55 percentage points over ShuffleNetv2, further substantiating the superiority of the enhancement method in striking a balance between precision and recall. This improvement in F1 score indicates that CSP-ML facilitates a more balanced strategy in managing false alarms and missed detections, which is pivotal for fire early warning systems as it enables a minimization of false positives and negatives and thereby enhances system reliability.

Finally, regarding inference speed, the CSP-ML-enhanced model achieved second place with an inference speed of 189 FPS; although it is 35 FPS behind ShuffleNetv2, it significantly outpaces the original DarkNet-53 model by 72 FPS. This demonstrates that CSP-ML, while substantially enhancing detection performance, does not introduce undue computational overhead, realizing an optimal balance between performance and efficiency. This efficient inference rate guarantees applicability in scenarios demanding high real-time performance, which underpins timely responses to fire emergencies.

In conclusion, evaluating the performance of various backbone network architectures indirectly illustrates the improved CSP-ML module’s prowess in processing deep-level feature information. While improvements at the data level are modest, they underscore the benefits of integrating the enhanced CSP-ML multi-level-feature-extraction network into the YOLOX model for mountain-fire-image-detection tasks. Future enhancements at the data level, combined with other improvements, have the potential to significantly elevate overall performance, affirming the effectiveness of comprehensive model optimization [48].

##### Analysis of Ablation Test Results

**Figure 11:**
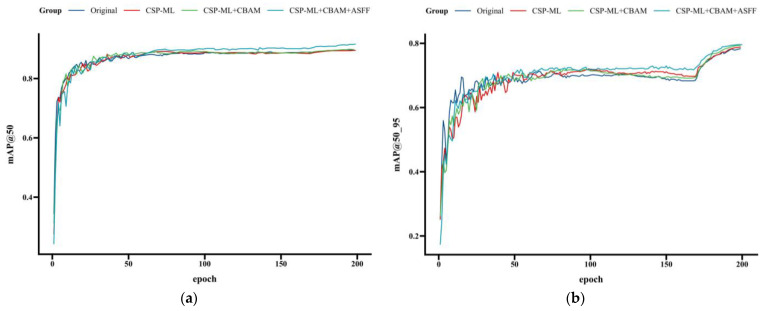
The ablation study demonstrates the changes in (**a**) mAP@50 and (**b**) mAP@50_95 with the integration of each module.

The curve shown in Figure 11 shows the trend of the mAP values after each added module is added, including mAP@50 and mAP@50_95. It is observable that the mAP values experience varying degrees of improvement with the successive addition of different modules, with the mAP values continuing to rise in the final unfreezing phase. From the close-up view, compared to the original YOLOX model, the improved network proposed in this study demonstrates more pronounced values for both mAP@50 and mAP@50_95, and the curves exhibit better convergence within the iterative cycles.

Additionally, Table 4 offers an insightful view into how the model’s performance evolves with the integration of different modules. With the original YOLOX model serving as a benchmark—featuring an mAP@50 of 89.3%, mAP@50_95 of 81.64%, FPS at 117 Hz, and parameter count at 8.94 M—the integration of the CSP-ML Multi-Level-Feature-Extraction Module into the model’s backbone network led to increases in mAP@50 and mAP@50_95 by 1.2 and 0.11 percentage points, respectively. This demonstrates CSP-ML’s enhanced capability to capture multi-scale features within fire images, thus improving the model’s detection accuracy for fire targets of diverse sizes. While the inclusion of CSP-ML raised the model’s parameter volume to 14.38 M, it notably enhanced overall performance, underscoring the significance of multi-level features in fire-detection tasks.

Upon this foundation, further integrating the CBAM attention mechanism led to increments of 0.1 and 0.62 percentage points in mAP@50 and mAP@50_95, respectively. These findings illustrate that CBAM, through adaptive adjustment of feature weights across spatial and channel dimensions, empowers the model to concentrate more intensely on significant regions and critical features of fire targets, mitigating background interference and enhancing detection precision. Remarkably, the CBAM module markedly enhances performance without a substantial increase in parameter count, highlighting its strengths in feature optimization. Although the incorporation of CBAM led to a minor reduction in FPS (to 176 Hz), it remains significantly above the original model, satisfying the model’s real-time performance criteria (exceeding 60 Hz).

Furthermore, the incorporation of the ASFF adaptive-feature-fusion module led to additional increases of 2.5 and 0.08 percentage points in the improved YOLOX model’s mAP@50 and mAP@50_95, respectively. This suggests that ASFF has the capability to adaptively modulate fusion weights in response to the significance of features across different scales, enabling a heightened focus on pertinent features within images, thereby augmenting detection precision. While the integration of ASFF marginally elevated the model’s complexity to 14.48 M, the significant performance gains achieved, along with the maintenance of a high FPS rate at 155 Hz, illustrate a harmonious balance between accuracy and speed.

Ultimately, substituting the loss function with the CIoU loss function led to further increments in the improved YOLOX model’s mAP@50 and mAP@50_95 by 2.6 and 1.36 percentage points, respectively, achieving an outstanding level of 96.3% and 83.81%. These findings demonstrate that the CIoU loss function, through its consideration of overlapping areas, center distances, and aspect ratios, offers more nuanced and detailed guidance for bounding box optimization, effectively hastening model convergence and augmenting detection accuracy. Furthermore, switching to the CIoU loss function did not augment the model’s parameter count or computational burden, thereby preserving the inference speed. This unequivocally underscores the CIoU loss function’s advantages in fine-grained bounding box optimization.

In conclusion, the integration of efficient feature-extraction and fusion mechanisms, including ASFF, CBAM, and CSP-ML, as well as the optimization of the loss function, has led to significant enhancements in the YOLOX model’s performance for mountain-fire-image-detection tasks. Although these enhancements marginally increased the model’s size and computational complexity, they preserved its status as a lightweight model. The findings demonstrate that the enhanced YOLOX model has struck an optimal balance among accuracy, efficiency, and embeddability, satisfying the requirements for high precision, rapid response, and deployment ease in initial wildfire-detection efforts. This underscores its potential for real-world application in mountain-fire surveillance, such as facilitating UAV-based wildfire patrols.

#### 4.4.3. Comparative Experiment

To further validate the enhanced performance of the improved YOLOX network in detecting mountain fires, representative single-stage-object-detection models (SSD and YOLOv5) and a two-stage-object-detection model (Faster R-CNN) were selected for comparative experiments. The results are presented in Table 5.

In conclusion, Table 5 reveals that the enhanced YOLOX model achieved an mAP of 96.3%, marking a 6.4% increase over the original YOLOX model (89.9%). This indicates that the improved model possesses stronger capabilities for detecting mountain-fire images and accurately identifying fire regions. Furthermore, the refined YOLOX model also demonstrates significant improvements in precision and recall rates. This underscores the model’s enhanced accuracy in predicting positive classes (fire regions) with a lower probability of false positives. This significantly reduces the likelihood of missed detections. Additionally, the detection speed of the enhanced YOLOX model significantly increased by 32.5% compared to the traditional YOLOX model, achieving 155 Hz. Compared to the Faster R-CNN model, the enhanced YOLOX exhibits significant enhancements across all performance metrics. Although Faster R-CNN shows better performance in recall rates, its precision and mAP are considerably lower than those of the enhanced YOLOX. Furthermore, Faster R-CNN has a relatively larger parameter count, lower real-time detection efficiency, and significantly higher computational complexity than other models. This renders it less practical in resource-constrained environments. Although the SSD model surpasses Faster R-CNN in terms of model size and computational complexity, its performance in mAP, precision, and recall rates falls below that of the enhanced YOLOX. YOLOv5 and the traditional YOLOX are closely matched in mAP, yet YOLOv5 falls short of the enhanced YOLOX in precision, recall rates, F1 scores, and real-time detection capabilities. However, YOLOv5 maintains a relatively lower parameter count and computational complexity, indicating higher detection efficiency at the expense of some detection performance. Although the computational complexity of the enhanced YOLOX model is slightly higher than that of the original YOLOX and YOLOv5 models, it remains significantly lower than that of Faster R-CNN and SSD models, making it well-suited for real-time mountain-fire-detection scenarios.

In summary, the enhanced YOLOX model exhibits exceptional performance in mountain-fire-image-detection tasks, outperforming comparative models in metrics such as mAP, precision, recall rates, F1 scores, and FPS. Although its model parameters and computational complexity are slightly higher than those of the original YOLOX and YOLOv5, it remains within the lightweight category, and the significant performance improvements attest to the efficacy of the modifications. While Faster R-CNN boasts a higher recall rate, its lower precision and substantial resource consumption limit its feasibility in practical applications. SSD and YOLOv5 perform well in terms of efficiency but fall short of the improved YOLOX model in precision and recall rates. Overall, the enhanced YOLOX model provides a well-balanced solution for mountain-fire detection, achieving high-precision target detection at a reasonable computational cost. This is crucial for the rapid and effective response and management of mountain fires. Future research could further explore how to reduce the model’s parameters and computational complexity while maintaining or even enhancing detection performance, to better adapt to resource-constrained practical application scenarios.

#### 4.4.4. Comparison of Scene Applications

**Figure 12:**
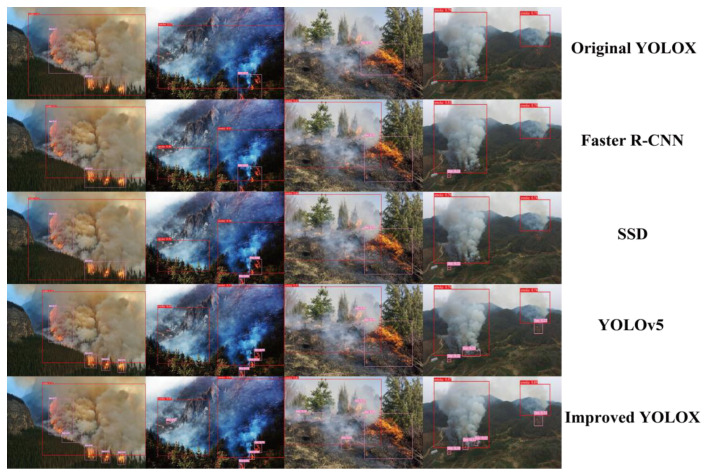
Comparison of different scene applications.

To more effectively illustrate the superiority of the enhanced YOLOX algorithm in mountain-fire detection, four sets of images from diverse scenarios were selected, including multiple-object scenes, complex dim scenes, complex bright scenes, and faint small-object scenes. Using five models, namely traditional YOLOX, Faster R-CNN, SSD, YOLOv5, and the enhanced YOLOX for the detection of mountain-fire targets in images, as demonstrated in Figure 12, the detection results of each algorithm include identification of flames and smoke, along with corresponding confidence scores, presented in the form of bounding boxes.

Initially, regarding multi-object mountain-fire-detection scenarios, the enhanced YOLOX model accurately detected all fire targets, including faint targets partially obscured by smoke, with high locational precision and a low rate of false alarms, demonstrating superior detection performance. In contrast, while the original YOLOX and YOLOv5 models could also detect most targets, they exhibited certain instances of missed detections. Faster R-CNN and SSD, however, significantly missed several targets, and showed broader target marking ranges, indicating poor detection performance. This suggests that the enhanced YOLOX model has stronger feature-extraction and target-association capabilities when handling multi-object complex scenes. Secondly, in the context of complex, dim fire backgrounds, all five models were able to effectively identify small fire points. Compared to the enhanced YOLOX model, the traditional YOLOX, Faster R-CNN, and SSD had larger recognition ranges with unclear boundaries. Although YOLOv5 could accurately locate targets, it had relatively lower confidence scores, and only the enhanced YOLOX model effectively captured tiny fire points on the image’s left side. Thirdly, for complex, bright fire scenes, the traditional YOLOX model lacked the ability to discern light smoke accompanying wildfires, which is failed to detect. In complex backgrounds with small fire points (including jungle coverage and light smoke), only the enhanced YOLOX model achieved effective recognition, while other models failed to capture them. Lastly, in scenarios of faint small-object mountain fires, all five models achieved effective detection of fire-smoke areas, with the enhanced YOLOX model maintaining relatively high confidence levels. For small fire areas, the traditional YOLOX, Faster R-CNN, and SSD models performed poorly not effectively marking small fires. YOLOv5 identified some small fire points, but compared to the enhanced YOLOX model it still had broader marking boundaries and reduced precision.

The comparative analysis demonstrates that the bounding boxes of the enhanced YOLOX model align more precisely with flames and smoke regions, thereby reducing the incorrect identification of non-fire areas. In contrast, other models, such as Faster R-CNN and SSD, produced broader or misaligned bounding boxes under complex conditions, which could lead to delayed or inaccurate responses to fires. Moreover, the enhanced YOLOX model consistently exhibited higher confidence scores than did the other models, indicating its superior reliability and an effective reduction in numbers of false-positive and false-negative results in practical applications. It is particularly noteworthy that the enhanced YOLOX model demonstrated superior performance in detecting faint small-object fire regions, which was exemplified in complex, dim backgrounds where only the enhanced YOLOX successfully marked small fire points that would facilitate early detection, which is crucial for preventing the spread of fires, while other models failed to detect such incidents. Overall, the enhanced YOLOX model surpasses the other four models in the accuracy of bounding box placement, consistency of confidence scores, and sensitivity to small-scale fires, showcasing a more pronounced improvement in comprehensive performance than is seen for the other models.

## 5. Discussion

1. Discussion of results

The autumn and winter seasons are periods of high frequency for mountain fires. Early warning and real-time detection of mountain fires are among the most crucial aspects of forest-protection efforts. This study introduces a lightweight, small-target-detection algorithm for mountain fires based on the improved YOLOX network and utilizing drone platforms to achieve rapid identification of wildfires. Ablation experiments reveal that optimizations to various modules significantly enhanced algorithm performance, affirming the effectiveness of these improvements. Through horizontal comparisons with models, including the original YOLOX, Faster R-CNN, SSD, and YOLOv5 models, the superiority of the improved YOLOX model in terms of accuracy and real-time performance in mountain-fire detection is validated. Comparisons of application-detection results in complex scenes demonstrate the advantages of the improved YOLOX in feature extraction, target classification, and background noise suppression.

2. Limitation analysis

Although the improved YOLOX model exhibits outstanding detection performance in the mountain-fire-detection task, it still has certain limitations and room for improvement. Firstly, the model utilizes a specific mountain-fire-image dataset during the training and testing process, and its generalization ability and adaptability need further validation. In the future, more mountain-fire-image data from different scenes and environments will be collected and tested to comprehensively evaluate the robustness of the improved model. Secondly, for complex mountain-fire recognition in areas with cloud and fog coverage in highlands or in dense forests with severe tree occlusion, the improved model still has some room for enhancement. Lastly, the mountain-fire-detection task not only requires the accurate localization of mountain-fire targets but it also necessitates the analysis and prediction of the severity and spreading trends of mountain fires. In the future, we will consider combining the improved YOLOX model with other techniques (such as semantic segmentation and trajectory prediction) to achieve more comprehensive and intelligent mountain-fire monitoring and early warning systems.

## 6. Conclusions

To achieve accurate and rapid identification of forest fires, this paper proposes a multi-scale fire-detection algorithm based on the improved YOLOX network that effectively addresses the issue of severe external interference in mountain-fire detection, which often leads to false alarms and missed detections. The main contributions of this study include the following achievements:

- Design of a multi-level-feature-extraction module, (CSP-ML): A novel multi-level-feature-extraction module, CSP-ML, was designed and integrated with the CBAM attention mechanism within the neck network. This effectively reduces background noise and enhances the detection accuracy of small-target-fire areas. Additionally, an adaptive feature-fusion module was introduced that utilizes the CIoU loss function to boost the network’s feature-learning capability and mitigate issues such as the excessive optimization of negative samples and poor gradient-descent direction. Compared to the traditional YOLOX network, this resulted in improvements of 6.4% in mAP@50 and 2.17% in mAP@50_95.
- Multi-scenario application testing: In tests involving multiple fire scenarios, such as multi-target flames and small-target flames, the improved YOLOX network demonstrated higher detection accuracy and stronger anti-interference capabilities than deep learning algorithms like Faster R-CNN, SSD, and YOLOv5. It proved to be suitable for detecting various forms of fire information in complex forest- and mountain-fire scenes, showcasing its strong practicality and high application value.

These advancements underline the potential of the improved YOLOX network in enhancing the efficiency and reliability of forest-fire-monitoring systems. By leveraging cutting-edge techniques in feature-extraction and attention mechanisms, along with the optimization of loss functions, the proposed solution offers a significant step forward in the intelligent detection of forest fires, which will contribute to more effective disaster-prevention and -response strategies.

## Figures and Tables

**Table 1:** The specific numbers of collected images.

Application Scenario	Quantity
Daylight	9080
Darkness	7737
Fire-like	1500
Smoke-like	2500

**Table 2:** Model size comparisons.

Model	Model Size	Classified as Lightweight Model	Model	Model Size	Classified as Lightweight Model
YOLOv1	753 MB	No	YOLOv6-N [31]	4.3 MB	Yes
YOLOv2	193 MB	No	YOLOv6-S [31]	15.0 MB	Yes
Tiny YOLOv2 [32]	60 MB	Yes	YOLOv6-M [31]	34.9 MB	Yes
YOLOv3	246 MB	No	YOLOv6-L	58.5 MB	No
Tiny YOLOv3 [33,34]	34 MB	Yes	YOLOv6-L-ReLU	58.5 MB	No
YOLOv4	245 MB	No	YOLOv7-Tiny [35]	6.2 MB	Yes
YOLOv4-Tiny [36,37]	23 MB	Yes	YOLOv7 [35]	36.9 MB	Yes
YOLOv5s [38,39]	14 MB	Yes	YOLOv7-X	71.3 MB	No
YOLOv5m [38,39]	42 MB	Yes	YOLOv7-W6	70.8 MB	No
YOLOv5l	90 MB	No	YOLOv7-E6	97.2 MB	No
YOLOv5x	168 MB	No	YOLOv7-D6	133.4 MB	No
YOLOX-Nano [40,41]	0.91 MB	Yes	YOLOv7-E6E	151.7 MB	No
YOLOX-Tiny [40,41,42]	5.06 MB	Yes	YOLOv8n [43,44]	6.1 MB	Yes
YOLOX-S [40,41,42,45]	9.0 MB	Yes	YOLOv8s [43,44,46]	21.6 MB	Yes
YOLOX-M [40,41,42,45,47]	25.3 MB	Yes	YOLOv8m	50.7 MB	No
YOLOX-L	54.2 MB	No	YOLOv8l	104.0 MB	No
YOLOX-X	99.1 MB	No	YOLOv8x	218.0 MB	No

**Table 3:** Comparison results for multi-level-feature-extraction structures.

Model	Backbone	Precision (%)	Recall (%)	mAP0.5 (%)	F1 Score (%)	FPS/Hz
YOLOX	DarkNet-53	93.89	93.69	89.9	93.97	117
YOLOX	ShuffleNetv2	87.65	85.34	76.58	78.96	224
YOLOX	Improved CSP-ML in DarkNet-53	94.21	93.97	91.1	94.51	189

**Table 4:** Ablation study results.

Network	mAP@50 (%)	mAP@50_95 (%)	FPS/Hz	Parameters/M
Original YOLOX	89.9	81.64	117	8.94
CSP-ML	91.1	81.75	189	14.38
CSP-ML + CBAM	91.2	82.37	176	14.40
CSP-ML + CBAM + ASFF	93.7	82.45	155	14.48
Improved YOLOX	96.3	83.81	155	14.48

**Table 5:** Comparative experiment results.

Network	mAP (%)	Precision (%)	Recall (%)	F1 Score	FPS/Hz	Parameters/M	Gflops/G
Original YOLOX	89.9	93.89	93.69	93.97	117	8.94	26.8
Faster R-CNN	62.8	46.2	72.47	56.33	65	137	185.1
SSD	88.6	78.38	93.75	85.33	72	26.29	140.9
YOLOv5	89.3	84.91	91.33	81	109	7.1	16.5
Improved YOLOX	96.3	95.33	94.94	94.13	155	14.4	35.2

## Data Availability

The data that support the findings of this study are available from the corresponding author upon reasonable request.
